# Waste-Glycerol-Directed Synthesis of Mesoporous Silica and Carbon with Superior Performance in Room-Temperature Hydrogen Production from Formic Acid

**DOI:** 10.1038/srep15931

**Published:** 2015-10-30

**Authors:** Dong-Wook Lee, Min-Ho Jin, Ji Chan Park, Chun-Boo Lee, Duckkyu Oh, Sung-Wook Lee, Jin-Woo Park, Jong-Soo Park

**Affiliations:** 1 Advanced Materials and Devices Laboratory, Korea Institute of Energy Research (KIER), 152 Gajeongro, Yuseong, Daejeon 305-343, Republic of Korea; 2 Clean Fuel Laboratory, Korea Institute of Energy Research (KIER), 152 Gajeongro, Yuseong, Daejeon 305-343, Republic of Korea

## Abstract

The development of easier, cheaper, and more ecofriendly synthetic methods for mesoporous materials remains a challenging topic to commercialize them, and the transformation of waste glycerol, as a biodiesel byproduct, into something useful and salable is one of the pending issues to be resolved. Here we first report that mesoporous silica (KIE-6) and carbon (KIE-7) can be simultaneously synthesized by using cheap and ecofriendly crude-waste-glycerol of biodiesel with or without glycerol purification, and we demonstrated the excellent performance of the mesoporous material as a catalyst support for formic acid decomposition. As a result, Pd-MnO_x_ catalysts supported on NH_2_-functionalized KIE-6 showed the highest catalytic activity (TOF: 540.6 h^−1^) ever reported for room-temperature formic acid decomposition without additives. Moreover, we conducted life-cycle assessment (LCA) from biomass cultivation through biodiesel production to KIE-6 and KIE-7 preparation, and it was confirmed that CO_2_ emission during synthesis of KIE-6 and KIE-7 could be reduced by 87.1% and 85.7%, respectively. We believe that our study suggested more ecofriendly and industry-friendly approaches for preparation of mesoporous materials, and utilization of waste glycerol.

Since surfactant-templated MCM-41 was first synthesized in 1992^1^,^2^, there has been intense research activity in synthesis of various mesoporous silica using diverse templates such as ionic^3^,^4^,^5^, neutral^6^,^7^,^8^,^9^ surfactants, and nonsurfactants^10^,^11^. However, ionic and neutral surfactants are expensive and not ecofriendly because of low biodegradability of their long hydrophobic organic segments. Moreover, replacement of surfactants by ones with different chain length and molecular weight, thermal aging for a long time, or use of toxic solvents as a swelling agent are required to expand the pore size. These disadvantages interrupt the applications of surfactant-templated mesoporous silica on an industrial scale. Therefore the development of more ecofriendly and economical synthetic pathways such as using nonsurfactants or surfactants from renewable resources remains a challenge in the field of mesoporous silica synthesis^12^.

Meanwhile, various mesoporous silica materials could be employed as a hard template for synthesis of non-siliceous mesoporous materials such as mesoporous carbon, titania and so on. Using MCM-48 as a hard template, Ryoo *et al.* and Hyeon *et al.* first prepared ordered mesoporous carbon in 1999^13^,^14^. Since then, a number of mesoporous silica materials with diverse mesostructures have been used for preparation of mesoporous carbon by a nanocasting method^15^,^16^,^17^,^18^. However, the nanocasting method needs multiple steps being composed of silica/template assembly formation, elimination of templates, carbon source filling, carbonization, and etching of silica framework. Such a complicated synthetic procedure is a major hindrance to the commercialization of the nanocasting method, and this opens a novel direct synthetic pathway of mesoporous carbon^19^, which involves the self-assembly of polymerizable carbon precursors and block copolymer templates^20^,^21^,^22^. Compared with the conventional nanocasting method, the direct synthesis method provides several advantages such as simpler procedures, lower cost, and suitability for industrial applications^19^. However, toxic carbon precursors such as phenol-formaldehyde, resorcinol-formaldehyde, and phloroglucinol-formaldehyde pairs^23^ are required for the formation of carbon scaffold and expensive and low-biodegradable block copolymers with different molecular weight^24^,^25^ are still necessary to control the pore size. Moreover, a large amount of CO_2_ is emitted during the high-temperature thermal treatment for the carbonization of carbon precursors and the thermal decomposition of surfactants as a templating agent. Thus, to commercialize such outstanding mesoporous materials such as silica, carbon, etc outside laboratories, easier, cheaper, and more ecofriendly synthetic methods for mesoporous materials is highly desired^12^.

Here we report that mesoporous silica and carbon can be synthesized by using cheap and ecofriendly glycerol as a biodiesel waste. Pure glycerol or crude waste glycerol without purification was employed as a pore-forming agent for mesoporous silica and as a carbon precursor for mesoporous carbon. After the glycerol-silica nanoparticle (G-SN) nanocomposites are precarbonized to obtain precarbonized glycerol-silica nanoparticle (PG-SN) nanocomposites, calcination of the PG-SN nanocomposite results in mesoporous silica designated as KIE-6 (Korea Institute of Energy research-6). In addition, when the G-SN nanocomposites are fully carbonized to form carbon-silica nanoparticle (C-SN) nanocomposites, followed by elimination of the silica framework from the C-SN nanocomposites, we can also synthesize mesoporous carbon replica denoted as KIE-7 (Korea Institute of Energy research-7). To the best of our knowledge, there are no precedents for the preparation of mesoporous silica and carbon using crude waste glycerol of biodiesel as a templating agent or a carbon precursor.

In particular, we used KIE-6 as a catalyst support for hydrogen production from formic acid decomposition at room temperature, which is of great importance for the development of low-temperature hydrogen storage and production systems, because formic acid has non-toxicity, high hydrogen content (53 g/L, 4.4 wt%), and availability of the existing gasoline and oil infrastructure. In case of formic acid decomposition with additives such as sodium formate, Xu *et al.*^26^ and Cai *et al.*^27^ recently reported the heterogeneous catalysts with excellent catalytic activity, which was comparable to homogeneous catalysts. However, in case of formic acid decomposition without additives, the catalytic activity of heterogeneous catalysts is still lower than that with additives. Thus, development of more efficient catalysts without additives remains a challenge. To verify the superior formic-acid-decomposition performance of KIE-6, we prepared the Pd-MnO_x_/NH_2_-KIE-6 catalyst, and compared it with other efficient catalysts^28^,^29^,^30^,^31^,^32^,^33^,^34^,^35^ reported by recent publications. Moreover, we calculated the CO_2_ reduction effect of KIE-6 and KIE-7 by utilizing waste glycerol as a template and a carbon precursor.

## Results

### Glycerol-directed mesoporous silica

**Figure 1:**
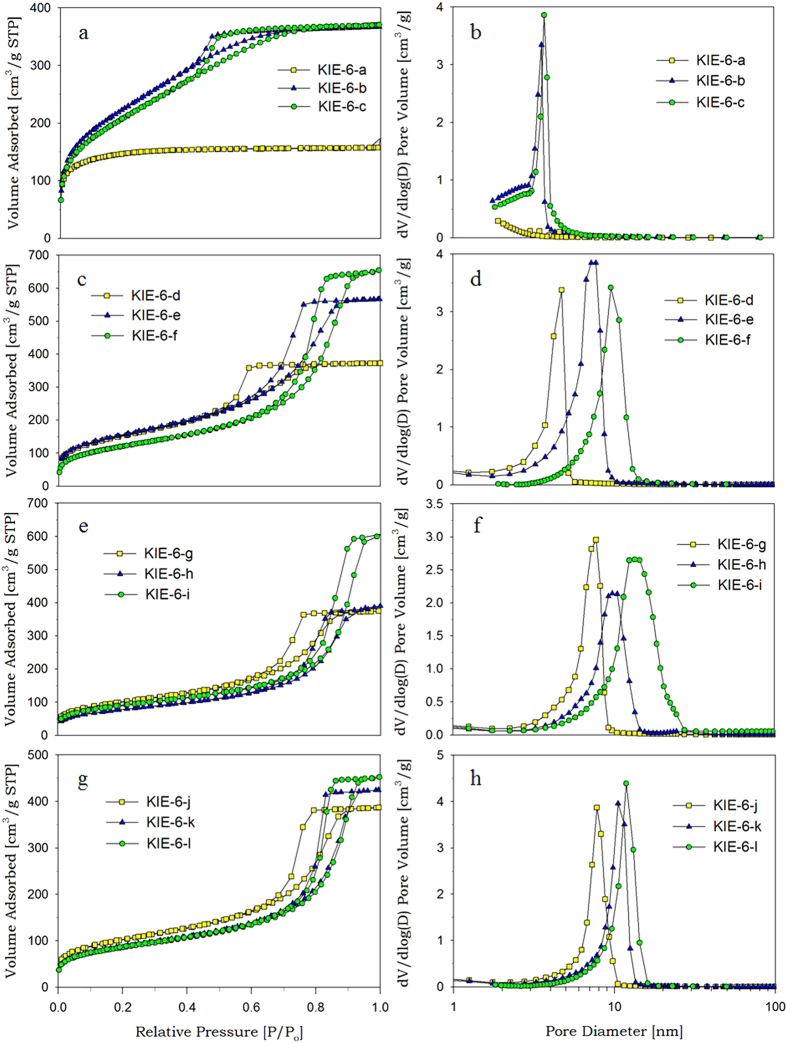
Nitrogen sorption results of KIE-6. (**a**,**b**) KIE-6-a, -b, -c. (**c,d**) KIE-6-d, -e, -f. (**e,f**) KIE-6-g, -h, -i. (**g,h**) KIE-6-j, -k, -l. (**a,c,e,g**) isotherms. (**b,d,f,h**) BJH desorption pore size distributions.

When mesoporous silica was prepared from G-SN nanocomposites, G-SN nanocomposites were slightly precarbonized at 150 °C to prevent the evaporation of glycerol before the formation of rigid silica framework through the condensation of surface silanol groups. Otherwise, glycerol is evaporated from G-SN nanocomposites before the condensation of silica nanoparticles, resulting in shrinkage of the volume occupied by glycerol. To verify the pore-controlling effect of glycerol, we synthesized various mesoporous silica with different pore properties, such as pore size, pore volume, specific surface area, and pore wall thickness of mesoporous silica, by changing the glycerol concentration of G-SN nanocomposite solution and varying the particle size of silica sol (polymeric, 7 nm, 11 nm, 15 nm). Table S1 shows sample codes for KIE-6 and KIE-7 prepared under different synthesis conditions, and Fig. 1 presents nitrogen sorption isotherms and pore size distributions for KIE-6 samples. As shown in Fig. 1a,b, when polymeric silica sol was dried and calcined without the addition of glycerol as a pore-forming agent, KIE-6-a gave a typical type I isotherm with no hysteresis loops and its peak pore diameter was below 2 nm, indicating that KIE-6-a prepared without glycerol provides microporous structure. However, for KIE-6-b and KIE-6-c prepared with glycerol as a pore-forming agent, they gave type IV isotherms with obvious H2 hysteresis loops, which demonstrates that mesoporous structure was successfully established by the elimination of precarbonized glycerol. As shown in the corresponding pore size distribution (Fig. 1b), pore size increased with an increase in the glycerol/silica ratio of G-SN nanocomposites. Their micropore surface area decreased from 533 m^2^/g to 198 m^2^/g, whereas BET surface area increased from 551 m^2^/g to 764–822 m^2^/g (Table S2), indicating that microporous structure was transformed into mesoporous structure due to the addition of glycerol templates.

**Figure 2:**
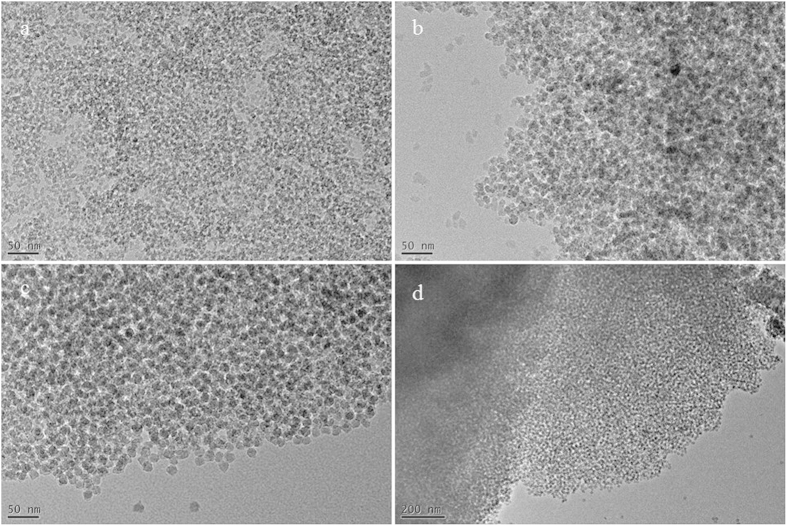
TEM images of KIE-6. (**a**) KIE-6-f, scale bar: 50 nm. (**b**) KIE-6-h, scale bar: 50 nm. (**c**) KIE-6-k, scale bar: 50 nm. (**d**) low magnification image for KIE-6-k, scale bar: 200 nm.

Using the same synthetic method as KIE-6-a, -b, and -c, we also synthesized further mesoporous silica samples only changing the nanoparticle size of silica sol (Table S1). Compared to polymeric silica sol, the xerogels (KIE-6-d and -g) prepared with colloidal silica sols with 5 nm, 11 nm, and 15 nm of particle size have essentially mesopore structure due to larger primary particle size than polymeric silica, even if glycerol as a pore-forming agent was not added to such colloidal silica sols. However, using glycerol as a pore-forming agent, we can easily expand the pore size and pore volume of mesoporous silica while its high surface area is maintained (Fig. 1c–h and Table S2). In the case of KIE-6-d, -e, and -f prepared with nanocomposites between 5 nm silica sol and glycerol, as the glycerol/silica weight ratio of G-SN nanocomposites increased from 0 through 4.9 to 8.0, the sharp inflection of hysteresis loops shifted toward higher P/P_o_ values and the corresponding peak pore size increased from 4.7 nm through 7.2 nm to 10.0 nm (Fig. 1c,d, and Table S2). Moreover, their pore volume dramatically increased from 0.52 cm^3^/g to 1.01 cm^3^/g, while the surface area was almost maintained at 443–550 m^2^/g (Table S2). As for KIE-6 samples prepared with 11 nm and 15 nm silica sols (KIE-6-d ~ -l), they also showed the same trend of pore size expansion as KIE-6-d ~ –f. Figure 2 exhibits TEM images of KIE-6 samples prepared with glycerol and colloidal silica sols with 5 nm, 11 nm, and 15 nm of particle size. In general, when silica nanoparticles form their aggregates without pore-forming agents, the nanoparticles are closely packed, minimizing their interstitial volume. However, as shown in Fig. 2, using glycerol as a pore-forming agents, all of the KIE-6-f, -h, and -k samples gave 3D-interconnected wormhole mesopore structure, expanding the interstitial volume among silica nanoparticles regardless of the silica nanoparticle size.

### Glycerol-directed mesoporous carbon

To synthesize mesoporous carbon from G-SN nanocomposites, we fully carbonized the G-SN nanocomposites at high temperature under nitrogen atmosphere, resulting in C-SN nanocomposites. After the silica-nanoparticle framework was eliminated from the C-SN nanocomposites through etching with NaOH solution, mesoporous carbon KIE-7 was successfully fabricated.

**Figure 3:**
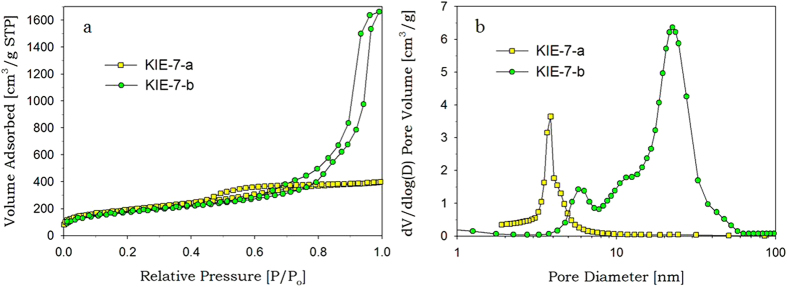
Nitrogen sorption results of KIE-7. (**a**) Isotherms. (**b**) BJH desorption pore size distributions.

**Figure 4:**
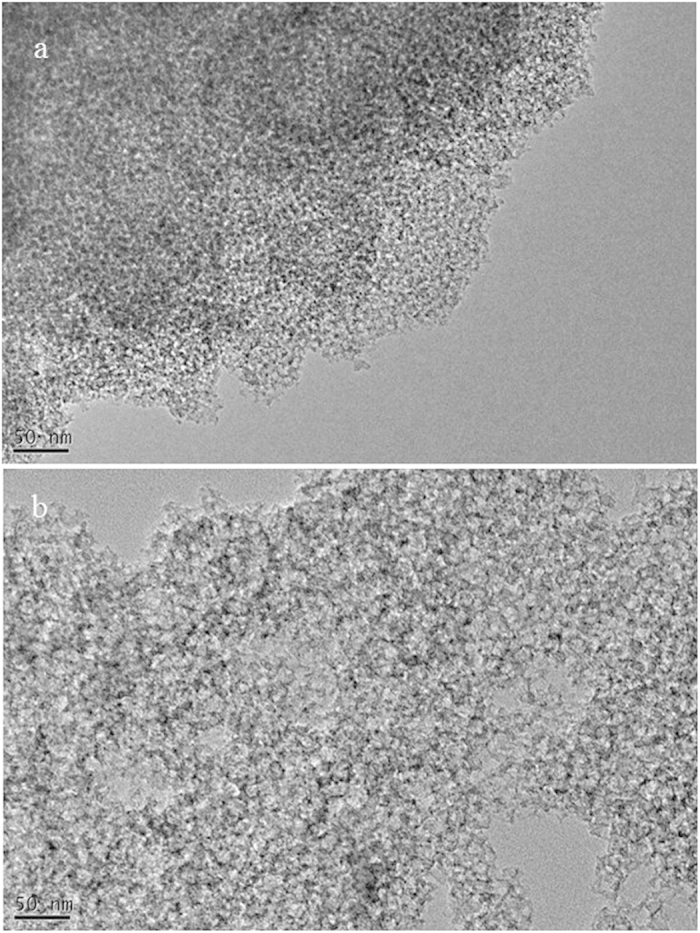
TEM images of KIE-7. (**a**) KIE-7-a, scale bar: 50 nm. (**b**) KIE-7-b, scale bar: 50 nm.

Our synthetic methods for mesoporous carbon can be classified into endo-hard templating, which is much more straightforward than conventional nanocasting (exo-hard templating) because of the absence of prior synthesis of mesoporous silica template, and is more ecofriendly in comparison with organic-organic self-assembly method (endo-soft templating) due to the exclusion of expensive and low-biodegradable block copolymer templates and toxic chemicals such as phenol and formaldehyde. Another advantage of KIE-7 is that the pore size and pore volume of mesoporous carbon can be readily tailored by simply changing the silica particle size in G-SN nanocomposites. As the silica particle size increased from 5 nm to 15 nm, the hysteresis loop of the isotherm considerably shifted toward higher P/P_o_, and adsorbed nitrogen volume significantly increased (Fig. 3a). The corresponding peak pore diameter shifted from 3.9 nm to 22.5 nm (Fig. 3b), and total pore volume remarkably increased from 0.62 cm^3^/g to 2.57 cm^3^/g (Table S2). Figure 4 shows TEM images of KIE-7-a and -b. It was revealed that KIE-7-a and -b gave the inverse-replica mesostructure of KIE-6-f and -k, respectively (Figs 2 and 4), and the increase of silica nanoparticle size boosted the pore size and pore volume of KIE-7.

### Synthesis of mesoporous silica and carbon with crude waste glycerol of biodiesel without purification

**Figure 5:**
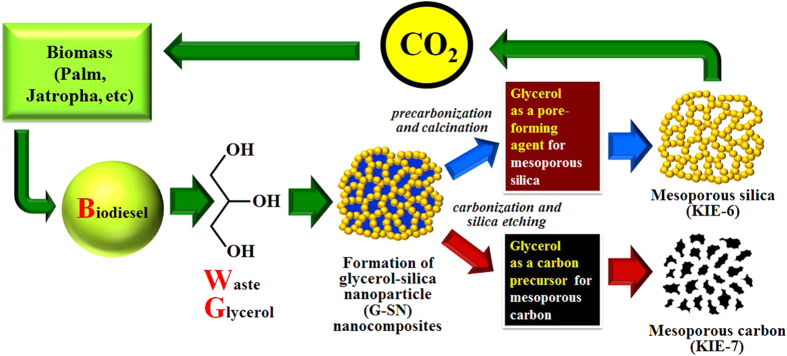
Schematic diagram for the synthesis mechanism of KIE-6 and KIE-7.

**Figure 6:**
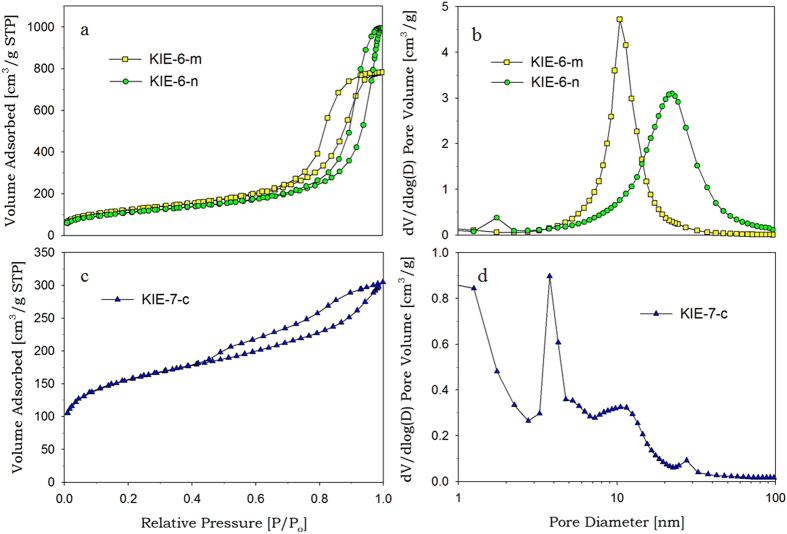
Nitrogen sorption results of KIE-6 and KIE-7 prepared with crude waste glycerol of biodiesel. (**a,b**) KIE-6-m, -n. (**c,d**) KIE-7-c. (**a,c**) isotherms. (**b,d**) BJH desorption pore size distributions.

Figure 5 is a schematic diagram for carbon neutral synthesis mechanism of mesoporous silica and carbon using biodiesel waste glycerol. Biodiesel is generally synthesized through transesterification of triglycerides extracted from biomass such as palm, jatropha, and so on. The transesterification of triglycerides produces 90 wt% of biodiesel and 10 wt% of glycerol as a byproduct. Every year, 1 million tons of glycerol byproduct are produced worldwide, and most of the glycerol byproduct is incinerated due to high costs for purification of crude glycerol. The simple incineration of crude glycerol emits 0.87 million tons of carbon dioxide. Thus, turning the glycerol byproduct into something useful remains a challenging topic^36^,^37^. We employed the glycerol byproduct as a pore-forming agent for the synthesis of mesoporous silica, as a carbon precursor for the synthesis of mesoporous carbon. Because carbon dioxide emitted during the synthesis process of mesoporous silica and carbon is absorbed again by biomass, we can achieve the carbon neutral cycle of glycerol-directed synthetic methods. To maximize the carbon dioxide reduction effect, crude waste glycerol is more favorable than purified glycerol. Accordingly, we tried to fabricate KIE-6 and KIE-7 using crude waste glycerol of biodiesel to confirm that crude waste glycerol also acts as a pore-forming agent of mesoporous silica and as a carbon precursor of mesoporous carbon. The crude waste glycerol used in this study is composed of water, methanol, glycerol, soap (alkaline salts of fatty acids), and fatty acid methyl esters (FAME). Glycerol, soap, and FAME account for 60 wt% of crude waste glycerol. Figure 6 exhibits nitrogen sorption isotherms and pore size distributions of KIE-6-m, -n, and KIE-7-c prepared with crude waste glycerol. In the case of KIE-6-m and -n, the hysteresis loops of isotherms shifted toward higher P/P_o_ (Fig. 6a), and the corresponding peak pore diameter and pore volume increased from 10.5 nm to 23.5 nm, from 1.21 cm^3^/g to 1.54 cm^3^/g with an increase in the addition amount of crude waste glycerol (Fig. 6b and Table S2). Compared to KIE-6 prepared with pure glycerol, KIE-6-m and -n gave much higher pore size and pore volume, because soap (alkaline salts of fatty acids) components might act as a pore-forming agent along with glycerol. It is considered that soap components in the crude waste glycerol bring a positive effect on expanding the pore diameter of mesoporous silica. As for mesoporous carbon prepared with crude glycerol, KIE-7-c gave multimodal pore size distribution consisting of micropores smaller than 2 nm, small mesopores centered at 3.8 nm, and large mesopores above 10 nm (Fig. 6d). The small mesopores with peak pore diameter of 3.8 nm is attributed to the elimination of silica-nanoparticle templates from the C-SN nanocomposites. The micropores and large mesopores are considered to be derived from major impurities in crude glycerol such as soap and FAME.

### Investigation on the pore size expansion effect of crude waste glycerol

**Figure 7:**
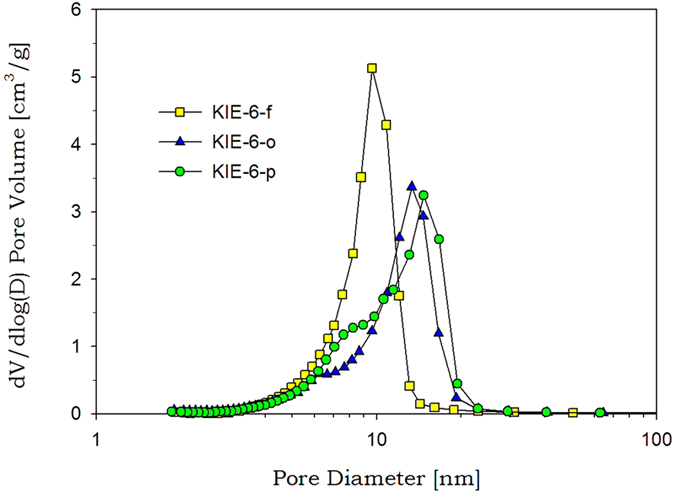
Pore size distributions of KIE-6-f, KIE-6-o, and KIE-6-p.

To study the cause of pore size expansion phenomena by crude waste glycerol, we prepared the simulated crude glycerol by adding sodium stearate (as alkaline salts of fatty acid) and diesel (as FAME) into glycerol solution. The mesoporous silica KIE-6-o was synthesized by adding sodium stearate into the pure-glycerol-based reactant solution for preparation of KIE-6-f, and the KIE-6-p was fabricated by adding both of sodium stearate and diesel (supplementary information). Figure 7 exhibits pore size distributions of KIE-6-f, KIE-6-o, and KIE-6-p. As a result, the peak pore diameter increased from 10.0 nm to 13.3 nm due to the addition of sodium stearate, and expanded from 10.0 nm to 14.7 nm by the addition of sodium stearate and diesel. Therefore it was confirmed that fatty acid salts and unseparated biodiesel (FAME), along with glycerol, included in crude waste glycerol could promote the pore size expansion of KIE-6.

### Catalytic activity of NH2-functionalized KIE-6 for room-temperature formic acid decomposition without additives

**Figure 8:**
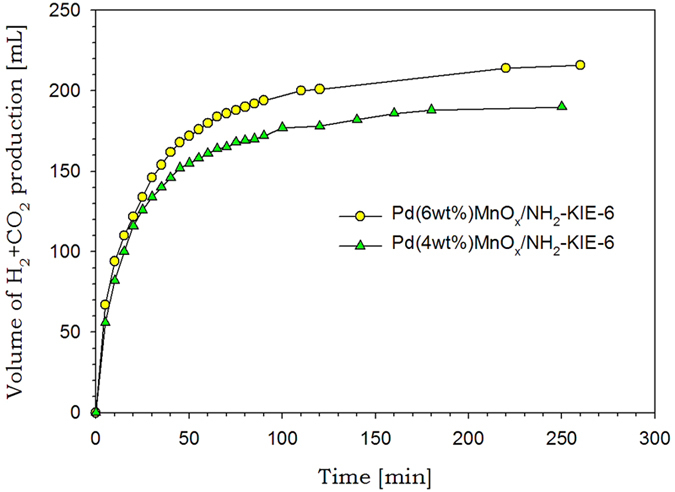
Formic acid decomposition activity of Pd-MnO_x_/NH_2_-KIE-6-f at 20 °C.

To verify the performance of KIE-6 as a catalyst support for hydrogen production from formic acid decomposition at room temperature without additives, we conducted the NH_2_-functionalization of KIE-6-f, and then prepared Pd(6wt%)-MnO_x_/NH_2_-KIE-6-f and Pd(4wt%)-MnO_x_/NH_2_-KIE-6-f as a formic acid decomposition catalyst. The metal loading contents for Pd(6wt%)-MnO_x_/NH_2_-KIE-6-f and Pd(4wt%)-MnO_x_/NH_2_-KIE-6-f were accurately estimated to be Pd(5.98wt%)/Mn(3.86wt%) and Pd(3.93wt%)/Mn(5.78wt%) respectively by inductively coupled plasma atomic emission spectroscopy (ICP-AES), which was almost consistent with the designed contents of metal loading. Figure 8 presents the plot of produced gas (H_2_ and CO_2_) volume versus the reaction time for Pd-MnO_x_/NH_2_-KIE-6-f catalysts. In the case of Pd(6wt%)-MnO_x_/NH_2_-KIE-6-f catalysts, 216 mL of H_2_ and CO_2_ was produced, and a turnover frequency (TOF) at initial 10 min and 20 °C was 405.9 mol H_2_ mol catalyst^−1^ h^−1^. For Pd(4wt%)-MnO_x_/NH_2_-KIE-6-f catalysts, 190 mL of H_2_ and CO_2_ was produced, and the catalysts provided 540.6 mol H_2_ mol catalyst^−1^ h^−1^ of TOF at initial 10 min and 20 °C, which is the highest catalytic activity ever reported for room-temperature formic acid decomposition without additives (Table S3)^28^,^29^,^30^,^31^,^32^,^33^,^34^,^35^. In addition, the volumetric ratio of H_2_ to CO_2_ was 50.3:49.7, and CO was not detected (detection limit <10 ppm).

Although we achieved superior catalytic activity in comparison with other publications shown in Table S3, it was very difficult to accurately compare the catalytic activity because of very diverse synthesis and test conditions. Thus we additionally prepared Pd(4wt%)/NH_2_-KIE-6-f, whose metal, support component, amine functionalization, and metal loading content are almost consistent with those of Pd(3.4wt%)/NH_2_-SBA-15 catalysts reported in the reference 31, and we conducted formic acid decomposition tests for the Pd(4wt%)/NH_2_-KIE-6-f catalysts at the same formic acid concentration and temperature as the reference 31. As a result, TOF (453.4 mol H_2_ mol catalyst^−1^ h^−1^) of the Pd(4wt%)/NH_2_-KIE-6-f catalysts was 1.54 times higher than that (293.0 mol H_2_ mol catalyst^−1^ h^−1^) of the Pd(3.4wt%)/NH_2_-SBA-15 (Table S3). Even though the metal, support component, amine functionalization, and metal loading content of the Pd(4wt%)/NH_2_-KIE-6-f were almost consistent with those of the Pd(3.4wt%)/NH_2_-SBA-15, the Pd(4wt%)/NH_2_-KIE-6-f gave much higher catalytic activity than Pd(3.4wt%)/NH_2_-SBA-15. Only one different factor between both catalysts is the pore structure of catalyst supports. KIE-6-f has much higher pore volume and larger pore size than SBA-15. In addition, KIE-6 has 3 dimensionally interconnected pore structure with good accessibility of reactants, whereas SBA-15 gives 2 dimensional hexagonal pore structure. Consequently, it is considered that the excellent catalytic activity of our catalysts was attributed to improved accessibility of formic acid to active sites. Moreover, mesoporous silica KIE-6 prepared by using glycerol as a pore-forming agent is expected to be a promising candidate for catalyst supports.

### Characterization of Pd-MnO_x_/NH_2_-KIE-6 catalysts

To increase the basicity of KIE-6 surface, 3-aminopropyl groups were functionalized on the surface of mesoporous silica KIE-6. The amine functionalization of KIE-6 was verified by fourier transform infrared (FTIR) spectra (Fig. S1). The peaks at 783 and 1026 cm^−1^ correspond to symmetric and asymmetric stretching vibration of Si-O-Si, respectively. The bands at 1547 and 2900 cm^−1^ are assigned to N-H bending and C-H stretching vibration in –(CH_2_)_3_NH_3_, which indicates the successful amine functionalization. When we prepare Pd-based catalysts supported on the amine-functionalized mesoporous silica, the amine functional groups generally provide several advantages for Pd-based formic acid decomposition catalysts. They facilitate the formation of small Pd nanoparticles and stabilize the as-prepared Pd nanoparticles. In addition, the basic amine functional groups promote the deprotonation of formic acid and the formation of Pd-formate. The electron transfer from the amine functional groups to Pd nanoparticles can be also induced by strong metal-support interaction (SMSI), which leads to better performance of formic acid decomposition^31^,^32^,^33^,^38^.

**Figure 9:**
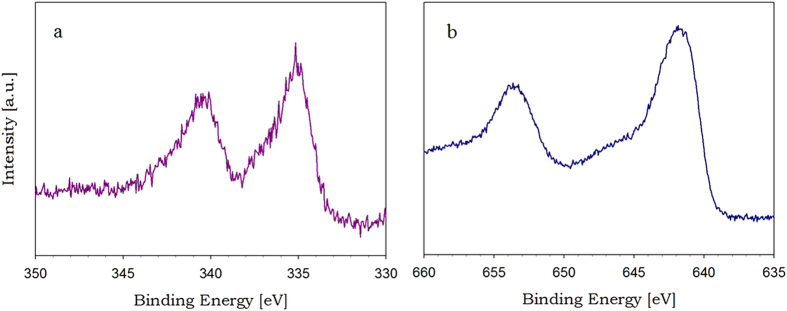
XPS spectra of Pd(4wt%)-MnO_x_/NH_2_-KIE-6-f. (**a**) Pd 3d spectra. (**b**) Mn 2p spectra.

**Figure 10:**
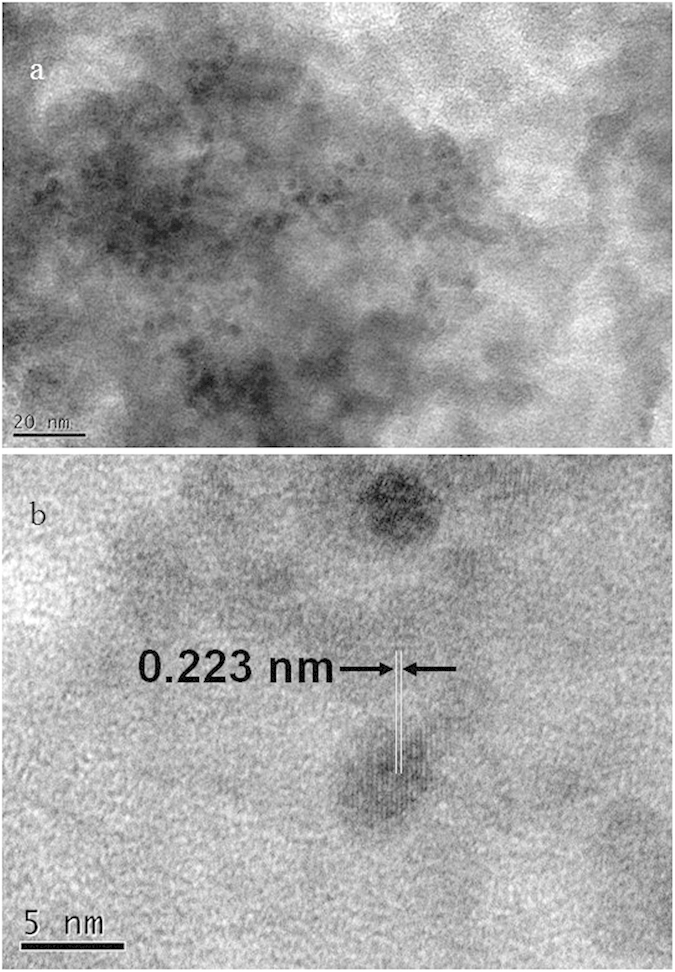
TEM images of Pd(4wt%)-MnO_x_/NH_2_-KIE-6-f. (**a**) low magnification image, scale bar: 20 nm. (**b**) high magnification image, scale bar: 5 nm.

The high electron density of Pd nanoparticles deposited on NH_2_-KIE-6-f was confirmed by the Pd 3d X-ray photoelectron spectroscopy (XPS) spectra (Fig. 9a). Intense peaks at 335.2 eV and 340.6 eV are ascribed to Pd 3d_5/2_ and Pd 3d_3/2_, and such low binding energy indicates the zero-valence metallic state of Pd nanoparticles with high electron density^33^. In addition, Fig. 9b exhibits XPS spectra of Mn 2p for the Pd-MnO_x_/NH_2_-KIE-6-f. The broad peak centered at 642.1 eV is associated with Mn 2p_3/2_ comprising Mn^2+^, Mn^3+^, and Mn^4+^ 32,33, demonstrating that the chemical state of Mn in our catalysts is the oxide state of MnO_x_. As shown in Fig. 10, the particle size of Pd and MnO_x_ nanoparticles was estimated to be 3.5–5.0 nm, and the lattice spacing distance of 0.223 nm was assigned to the (111) planes of Pd nanocrystalline, whereas the lattice fringes of MnO_x_ were not detected. In addition, the X-ray maps in Fig. S2 demonstrated the well distribution of Pd and MnO_x_ nanoparticles. On the basis of Fig. 10 and S2, it was revealed that Pd nanocrystal particles and amorphous MnO_x_ nanoparticles were physically mixed and deposited on NH_2_-KIE-6-f supports. In conclusion, taking into account all of the data on catalytic activity and characterization of Pd-MnO_x_/NH_2_-KIE-6, we deduce that the superior performance of NH_2_-KIE-6-f supported Pd catalysts originated from large pore size of KIE-6-f, basic amine sites on KIE-6-f, and high electron density of Pd induced by the electron transfer from amine groups.

### CO_2_ reduction effects of KIE-6 and KIE-7

Meanwhile, synthesis processes for mesoporous materials are generally energy-consuming and high-carbon-emission processes, because high-temperature thermal treatments such as calcination and carbonization are essential procedures. Therefore the development of low-carbon-emission synthetic processes is one of the pending issues to be resolved before their commercialization. To investigate CO_2_ reduction effects of KIE-6 and KIE-7 by using biodiesel-waste glycerol (Fig. 5), we conducted life-cycle assessment (LCA) from biomass cultivation through biodiesel production to KIE-6-k and KIE-7-b preparation (supplementary information). As a result, it was confirmed that CO_2_ emission during synthesis of KIE-6-k and KIE-7-b could be reduced by 87.1% and 85.7%, respectively. We believe that our study suggested more ecofriendly and industry-friendly approaches for preparation of mesoporous materials. However, further study is required to fully understand the pore formation mechanism by crude waste glycerol and to find additional applications of KIE-6 and KIE-7.

## Discussion

Mesoporous silica and carbon were synthesized by using pure glycerol or crude waste glycerol of biodiesel. In the case of mesoporous silica KIE-6, pore properties such as pore size, pore volume, specific surface area, and pore wall thickness can be controlled by changing glycerol concentration of G-SN nanocomposites or changing the particle size of silica sol. As for mesoporous carbon KIE-7, its pore size and pore volume can be readily tailored by simply changing the silica particle size in G-SN nanocomposites. Particularly, Pd-MnO_x_ catalysts supported on NH_2_-KIE-6 gave much higher catalytic performance for formic acid decomposition in comparison with other excellent catalysts reported by recent publications, which demonstrated the availability of KIE-6 as a catalyst support.

Moreover, the life-cycle assessment (LCA) during synthesis of KIE-6 and KIE-7 demonstrated that CO_2_ emission amount during the synthesis of KIE-6 and KIE-7 was much lower than the existing synthetic methods for mesoporous silica and carbon, and the CO_2_ reduction effect was attributed to the utilization of renewable resource as a templating agent and a carbon precursor for the synthesis of mesoporous silica and carbon.

In summary, using our new synthetic method, we significantly reduced the CO_2_ emission during preparation of mesoporous materials and excluded expensive and low-biodegradable surfactants from the synthesis process. Taking into account these achievements, even though crude waste glycerol includes toxic methanol as a reactant for production of biodiesel, our synthetic methods for mesoporous silica and carbon are considered to be more ecofriendly and economical than the existing synthetic methods such as surfactant-templating methods, nanocasting, etc. Nevertheless, because methanol included in crude glycerol is obviously one of obstacles to the development of green synthetic methods, the toxic methanol should be eliminated from crude glycerol. However, high purity glycerol is not required, because water, soap, and FAME included in crude glycerol is allowed to be used in our synthesis process. Thus, in near future, further research is required to explore the preparation of methanol-free crude glycerol and its application to synthesis of mesoporous materials.

## Methods

### Preparation of mesoporous silica KIE-6

Mesoporous silica KIE-6 was fabricated via formation of glycerol-silica nanoparticle (G-SN) nanocomposites, precarbonization, and calcination. As a pore-forming agent for KIE-6, we used pure glycerol (99%, DUKSAN) or crude waste glycerol of biodiesel (water and methanol: 40wt%; Glycerol, soap, and fatty acid methyl esters (FAME): 60 wt%). In a typical synthesis, glycerol and sulfuric acid (3 wt% of glycerol) were added into silica sol, and the mixture was vigorously stirred for 10 min at room temperature to form G-SN nanocomposite sol. The G-SN nanocomposite sol was dried and precarbonized at 150 °C for 12 h in air. Subsequently, the precarbonized glycerol-silica nanoparticle (PG-SN) nanocomposites were calcined at 550 °C for 2 h in air, resulting in mesoporous silica KIE-6. The detailed synthesis conditions for various KIE-6 samples were shown in Table S1.

### Preparation of mesoporous carbon KIE-7

Mesoporous carbon KIE-7 was fabricated via formation of glycerol-silica nanoparticle (G-SN) nanocomposites, carbonization, and etching of silica templates. In a typical synthesis, the PG-SN nanocomposites prepared for KIE-6 were fully carbonized at 600 °C for 3 h under nitrogen atmosphere, leading to formation of carbon-silica nanoparticle (C-SN) nanocomposites. After elimination of silica-nanoparticle framework from C-SN nanocomposites through etching with 5 wt% NaOH solution at 75 °C, mesoporous carbon KIE-7 was successfully prepared. The detailed synthesis conditions for various KIE-7 samples were shown in Table S1.

### Preparation of Pd-MnO_x_/NH_2_-KIE-6 catalysts for formic acid decomposition

As a formic-acid-decomposition catalyst, we prepared Pd(6wt%)-MnO_x_/NH_2_-KIE-6 and Pd(4wt%)-MnO_x_/NH_2_-KIE-6. In a typical synthesis, 0.1 g of KIE-6-f was added into 30 mL of toluene, followed by addition of 0.25 mL of 3-aminopropyl trimethoxysilane (APTMS: Aldrich, 97%). The final mixture was refluxed without stirring for 3 h at 110 °C. After filtering and washing unreacted APTMS with toluene, the NH_2_-functionalized KIE-6-f was successfully prepared. Subsequently, 0.029 g of manganese(II) chloride tetrahydrate (Aldrich) and 0.12 g of 10wt % palladium(II) nitrate aqueous solution (PM RESEARCH) were added into 10 mL of distilled water, followed by dispersion of 0.18 g of NH_2_-KIE-6-f. Afterward, 2 mL of 0.85M NaBH_4_ aqueous solution was added into the mixture solution, followed by stirring for 1 h. After centrifugation, washing, and drying, the Pd(6wt%)-MnO_x_/NH_2_-KIE-6-f was successfully prepared. For the preparation of Pd(4wt%)-MnO_x_/NH_2_-KIE-6-f, synthesis procedures were the same as those of the Pd(6wt%)-MnO_x_/NH_2_-KIE-6-f except that 0.043 g of manganese(II) chloride tetrahydrate and 0.08 g of 10wt % palladium(II) nitrate aqueous solution were added into 10 mL of distilled water.

### Preparation of Pd/NH_2_-KIE-6 catalysts for formic acid decomposition

In a typical synthesis, 0.075 g of 10wt % palladium(II) nitrate aqueous solution (PM RESEARCH) were added into 10 mL of distilled water, followed by dispersion of 0.18 g of NH_2_-KIE-6-f. Afterward, 2 mL of 0.85M NaBH_4_ aqueous solution was added into the mixture solution, followed by stirring for 1 h. After centrifugation, washing, and drying, the Pd(4wt%)/NH_2_-KIE-6-f was successfully prepared.

### Catalytic Decomposition of Formic Acid at Room Temperature

0.055g of Pd-MnO_x_/NH_2_-KIE-6-f (or Pd/NH_2_-KIE-6-f) was sealed in a 100mL Teflon-lined reactor, followed by a nitrogen purge for 30 min. After the nitrogen purge, the outlet of the reactor was connected to a gas burette system filled with water. Subsequently, a mixture of 10 mL of distilled water and 0.19 mL (or 0.38 mL) of formic acid (95%, Aldrich) was injected into the reactor through a rubber septum. The volume of gas generated at 20 °C (or 25 °C) was measured by the gas burette system.

### Characterization

The pore properties of KIE-6 and KIE-7 were taken by nitrogen sorption tests with a Micromeritics ASAP 2420 instrument. Degassing of samples was conducted at 200 °C for 5 h. Transmission electron microscopy (TEM) analyses were conducted by using a FEI/TECNAI G2 instrument. For characterization of the Pd(4wt%)-MnO_x_/NH_2_-KIE-6-f catalyst, we conducted inductively coupled plasma atomic emission spectroscopy (ICP-AES), X-ray photoelectron spectroscopy (XPS), fourier transform infrared (FTIR), TEM analyses. ICP-AES and XPS analyses were performed using Thermo Scientific iCAP 6500 and a Kratos 165XP spectrometer, respectively. FTIR analysis was carried out by using a Thermo Nicolet 5700 instrument.

## Additional Information

**How to cite this article**: Lee, D.-W. *et al.* Waste-Glycerol-Directed Synthesis of Mesoporous Silica and Carbon with Superior Performance in Room-Temperature Hydrogen Production from Formic Acid. *Sci. Rep.* **5**, 15931; doi: 10.1038/srep15931 (2015).

## Supplementary Material

Supplementary Information
