# Supplementary material for: Waste-Glycerol-Directed Synthesis of Mesoporous Silica and Carbon with Superior Performance in Room-Temperature Hydrogen Production from Formic Acid
Source: Sci Rep. 2015 Oct 30;5:15931. doi: 10.1038/srep15931 (PMC4626865; doi:10.1038/srep15931)
Supplement: Supplementary Information [file srep15931-s1.doc]

**Supplementary Information**

**Waste-Glycerol-Directed Synthesis of Mesoporous Silica and Carbon with Superior Performance in Room-Temperature Hydrogen Production**

**from Formic Acid**

**Dong-Wook Lee1,*, Min-Ho Jin1, Ji Chan Park2, Chun-Boo Lee1, Duckkyu Oh1,**

**Sung-Wook Lee1, Jin-Woo Park1, and Jong-Soo Park1**

**1** Advanced Materials and Devices Laboratory, Korea Institute of Energy Research (KIER),

*152 Gajeongro, Yuseong, Daejeon 305-343, Republic of Korea,*

**2** Clean Fuel Laboratory, Korea Institute of Energy Research (KIER),

*152 Gajeongro, Yuseong, Daejeon 305-343, Republic of Korea*

** corresponding author: dwlee99@kier.re.kr*

**1. Experimental**

**Preparation of Silica Sols.** Polymeric silica sol and colloidal silica sol with different particle diameter were synthesized to employ them as a framework for mesoporous silica and as a hard template for mesoporous carbon. Polymeric silica sol was synthesized under acid catalyzed condition. In a typical synthesis, for prehydrolysis of 0.1 mol of tetraethyl orthosilicate (TEOS: 98%, Aldrich), a mixture of TEOS, distilled water, and nitric acid (molar ratio=1:5.8:0.083) was vigorously stirred at room temperature, and then additional distilled water was added into the mixture to adjust the volume to 500 mL. Subsequently, the mixture was refluxed at 80 oC for 8 h, resulting in transparent polymeric silica sol with particle diameter below 2 nm.

Colloidal silica sol with particle diameter of about 5 nm was synthesized under base catalyzed condition. A NH3/H2O mixture was added into a TEOS/EtOH mixture vigorously stirred at 50 oC to adjust the final molar ratio of TEOS:NH3:H2O:EtOH to 1:0.086:53.6:40.7. Afterward the final mixture solution was refluxed for 3 h to obtain transparent colloidal silica sol. Colloidal silica sol with particle diameter of about 11 nm was synthesized through the same method as the 5 nm-silica sol except for TEOS:NH3:H2O:EtOH molar ratio of 1:0.36:53.6:40.3. In addition, colloidal silica sol with particle diameter of about 15 nm was also prepared with TEOS:NH3:H2O:EtOH molar ratio of 1:0.0084:19.8:2.2. The synthesis procedures were the same as the 5 nm-silica sol except for refluxing for 3 days.

**Preparation of mesoporous silica KIE-6-o and KIE-6-p with simulated crude glycerol.** To investigate the cause of pore size expansion phenomena by crude waste glycerol, we prepared the simulated crude glycerol by adding sodium stearate (as alkaline salts of fatty acid) and diesel (as FAME) into glycerol solution. The mesoporous silica KIE-6-o was synthesized by adding sodium stearate into the pure-glycerol-based reactant solution for preparation of KIE-6-f, and the KIE-6-p by adding both of sodium stearate and diesel. In a typical synthesis of KIE-6-p, 4 g of glycerol and sulfuric acid (3 wt% of glycerol) were added into 50 mL of silica sol, and the mixture was vigorously stirred for 10 min at room temperature to form G-SN nanocomposite sol. 2 g of sodium stearate (JUNSEI) was dissolved in 20 mL of methanol, and the sodium stearate solution was added into the G-SN nanocomposite, followed by the addition of 3.72 g of diesel fuel. The final mixture solution was dried at 150 oC for 12 h in air. Subsequently, the dried samples were calcined at 550 oC for 2 h in air, resulting in mesoporous silica KIE-6-p. The synthesis procedure for KIE-6-o is the same as that for KIE-6-p except that diesel fuel was not added.

**2. Supporting tables and figures**

Table S1. Synthesis conditions of KIE-6 and KIE-7 samples.

| Sample code | Silica framework | Glycerol  type | Glycerol/silica  weight ratio | Template elimination method |
| --- | --- | --- | --- | --- |
| KIE-6-a | polymeric silica | pure | 0 | calcination |
| KIE-6-b | polymeric silica | Pure | 5.1 | calcination |
| KIE-6-c | polymeric silica | Pure | 8.1 | calcination |
|  |  |  |  |  |
| KIE-6-d | 5nm silica | pure | 0 | calcination |
| KIE-6-e | 5nm silica | pure | 4.9 | calcination |
| KIE-6-f | 5nm silica | pure | 8.0 | calcination |
|  |  |  |  |  |
| KIE-6-g | 11nm silica | pure | 0 | calcination |
| KIE-6-h | 11nm silica | pure | 6.9 | calcination |
| KIE-6-i | 11nm silica | pure | 19.8 | calcination |
|  |  |  |  |  |
| KIE-6-j | 15nm silica | pure | 1.4 | calcination |
| KIE-6-k | 15nm silica | pure | 2.1 | calcination |
| KIE-6-l | 15nm silica | pure | 4.0 | calcination |
|  |  |  |  |  |
| KIE-6-m | 5nm silica | biodiesel waste | 0.6a | calcination |
| KIE-6-n | 5nm silica | biodiesel waste | 1.2a | calcination |
|  |  |  |  |  |
| KIE-7-a | 5nm silica | pure | 2.1 | carbonization and etching |
| KIE-7-b | 15nm silica | pure | 2.1 | carbonization and etching |
|  |  |  |  |  |
| KIE-7-c | 5nm silica | biodiesel waste | 1.2a | carbonization and etching |

a (glycerol + soap + FAME)/silica weight ratio

Table S2. Pore properties for KIE-6 and KIE-7 obtained from nitrogen sorption tests.

| Sample code | SABET  [m2/g]a | SAmicro  [m2/g]b | Vtot  [cm3/g]c | D  [nm]d |
| --- | --- | --- | --- | --- |
| KIE-6-a | 551 | 533 | 0.27 | 2.4 |
| KIE-6-b | 822 | 304 | 0.57 | 2.8 |
| KIE-6-c | 764 | 198 | 0.57 | 3.0 |
|  |  |  |  |  |
| KIE-6-d | 540 | 35 | 0.52 | 3.8 |
| KIE-6-e | 550 | 54 | 0.88 | 5.6 |
| KIE-6-f | 443 | 20 | 1.01 | 8.7 |
|  |  |  |  |  |
| KIE-6-g | 356 | 38 | 0.58 | 5.8 |
| KIE-6-h | 277 | 31 | 0.61 | 7.9 |
| KIE-6-i | 320 | 53 | 0.94 | 10.9 |
|  |  |  |  |  |
| KIE-6-j | 359 | 65 | 0.60 | 6.4 |
| KIE-6-k | 309 | 59 | 0.66 | 8.2 |
| KIE-6-l | 313 | 71 | 0.70 | 10.0 |
|  |  |  |  |  |
| KIE-6-m | 433 | 50 | 1.21 | 9.8 |
| KIE-6-n | 387 | 52 | 1.54 | 14.3 |
|  |  |  |  |  |
| KIE-7-a | 685 | 87 | 0.62 | 3.8 |
| KIE-7-b | 612 | 103 | 2.57 | 14.8 |
|  |  |  |  |  |
| KIE-7-c | 545 | 328 | 0.4 | 4.5 |

a BET surface area

b micropore surface area calculated from a t-plot

c total pore volume taken from the volume of nitrogen adsorbed at P/Po=0.995

d BJH desorption average pore diameter

Table S3. Catalytic activity comparison of various heterogeneous catalysts for room-temperature formic acid decomposition without additives.

| Catalysts | Formic acid concentration  [mmol] | additives | Reaction temperature [oC] | TOF  at 10 min a  [h-1] | Reference b |
| --- | --- | --- | --- | --- | --- |
| Pd(4wt%)-MnOx  /NH2-KIE-6 | 5 | - | 20 | 540.6 | This work |
| Pd(6wt%)-MnOx  /NH2-KIE-6 | 5 | - | 20 | 405.9 | This work |
| Pd(4wt%)  /NH2-KIE-6 | 10 | - | 25 | 453.4 | This work |
| Ag@Pd(10wt%)  /Vulcan carbon | 10 | - | 20 | 26.0 | [28] |
| Co(3wt%)Au(3.5wt%)Pd(3.5wt%)/Vulcan XC-72 | 5 | - | 25 | 80.0 | [29] |
| Pd(8wt%)/CN | 10 | - | 15 | 71.0 | [30] |
| Pd(3.4wt%)  /NH2-SBA-15 | 10 | - | 26 | 293.0 | [31] |
| Pd(1.82wt%)-MnOx  /NH2-silica gel | 2.65 | - | 20 | 248.7 | [32] |
| Au(4.6wt%)  Pd(2.8wt%)-MnOx  /ZIF-8-rGO | 5 | - | 25 | 382.1 | [33] |
| Pd(38.7wt%)  Ag(36.3wt%)/MIL-100 | 10 | - | 25 | 20.5 | [34] |
| Pd(4.6wt%)  Ag(9.3wt%)/TiO2 | 5 | - | 27 | 190.1 | [35] |

a TOF was calculated at initial 10 min of reaction time by using this equation (TOF=mole of produced hydrogen gas per hour/mole of metal catalyst). b see the references in the main text.

Figure S1. FTIR spectrum for NH2-functionalized KIE-6.

Figure S2. X-ray maps for selected area of Pd(4wt%)-MnOx/NH2-KIE-6.

**3. Calculation of net CO2 reduction through a green synthetic methodology of mesoporous silica KIE-6 using glycerol-silica(G-SN) nanoparticle nanocomposites**

**3.1. Calculation of CO2 emission during synthesis of KIE-6-k**

When the total weight of G-SN nanocomposite sol is set to 27.28 g, the nanocomposite sol consists of 2.52 g of silica nanoparticles, 15 g of distilled water, 4.26 g of ethanol, and 5.5 g of glycerol. The first step of KIE-6-k synthesis is drying and precarbonization of the nanocomposite sol at 150 oC. The energy required for thermal treatment of G-SN nanocomposite sol 27.28 g at 150 oC can be calculated as below.

Q150 = (Wsn × Csn × (150-25) + Wg × Cg × (150-25) + Ww × Cw × (100-25) + Ww × Qw

+ We × Ce × (78.37-25) ­+ We × Qe) + (Wsn × Csn + Wg × Cg) × HL × t150

= (2.52 × 0.22 × (150-25) + 5.5 × 0.58 × (150-25) + 15 × 1 × (100-25) + 15 × 539

+ 4.26 × 0.54 × (78.37-25) ­+ 4.26 × 229) + (2.52 × 0.22 + 5.5 × 0.58) × 100 × 12

= 15,269.6 cal

where Q150 : energy required for thermal treatment of G-SN nanocomposites at 150 oC,

Wsn, Wg, Ww, We : weight of silica nanoparticles, glycerol, water, and ethanol (g),

Csn, Cg, Cw, Ce : specific heat of silica nanoparticles, glycerol, water, and ethanol (cal/g.oC),

Qw, Qe : heat of vaporization of water, and ethanol (cal/g),

HL : heat loss rate of an electric furnace (assumption: 100 oC/h),

t150 : thermal treatment time at 150 oC (12 h)

We measured that the weight of G-SN nanocomposites was reduced from 27.28 g to 4.56 g after the thermal treatment at 150 oC, and 4.56 g of precarbonized glycerol-silica nanoparticle (PG-SN) nanocomposites comprises 2.52 g of silica nanoparticles and 2.04 g of precarbonized glycerol. The PG-SN nanocomposites were calcined at 550 oC for 2 h. If precarbonized glycerol is assumed to be carbon (because of complex components of precarbonized glycerol) and the carbon is considered to be completely eliminated at 550 oC, we can calculate the energy required for calcination of PG-SN nanocomposites at 550 oC for 2 h as below.

Q550 = (Wsn × Csn × (550-25) + Wc × Cc × (550-25)) + (Wsn × Csn) × HL × t550

= (2.52 × 0.22 × (550-25) + 2.04 × 0.17 × (550-25)) + (2.52 × 0.22) × 100 × 2

= 584.0 cal

where Q550 : energy required for calcination of PG-SN nanocomposites at 550 oC,

Wsn, Wc : weight of silica nanoparticles and carbon (g),

Csn, Cc : specific heat of silica nanoparticles and carbon (cal/g.oC),

HL : heat loss rate of an electric furnace (assumption: 100 oC/h),

t550 : calcination time at 550 oC (2 h)

If we ignore the thermal efficiency of the electric furnace and the energy required for heating air inside the furnace and ignore the weight change of silica by condensation during calcination, total energy consumption and CO2 emission per 1 g of the prepared KIE-6-k can be conservatively calculated as below:

Q = (Q150 + Q550)/WKIE-6-k = (15,269.6 + 584.0)/2.52 = 6,291.1 cal/g KIE-6-k

where WKIE-6-k : weight of KIE-6-k after calcination

WCO2,6k-I = 6,291.1 / 1000 × 0.001163 × 0.424 = 0.0031 kg CO2/g KIE-6-k

where WCO2,6k-I : CO2 emission during synthesis of KIE-6-k,

0.001163: unit conversion factor (kWh/kcal),

0.424: CO2 emission coefficient (kg CO2/kWh)

**3.2. Calculation of CO2 reduction by biodiesel-waste glycerol used for synthesis of KIE-6-k**

Nanaki et al.1 reported Life Cycle Assessment (LCA) of rapeseed oil-based biodiesel in Greece. According to their publication, when biodiesel is employed for transportation, CO2 emission amount is reduced from 134 g/km to 7.4 g/km in comparison with diesel. Using these previous data reported by Nanaki et al., we roughly calculated the CO2 reduction amount per unit weight of produced biodiesel as below.

WCO2,6k-II = (134 – 7.4) / 0.051 / 1000 / 0.88 = 2.82 kg CO2/kg biodiesel

where WCO2,6k-II : CO2 reduction amount per unit weight of produced biodiesel,

0.051: fuel efficiency of diesel vehicle (L/km),

0.88: biodiesel density (kg/L) 2

The CO2 reduction amount per unit weight of produced glycerol is calculated as below.

WFgc = (0.09 × 0.391) / (0.09 × 0.391 + 0.91 × 0.861) = 0.043

where WFgc : weight fraction of glycerol-derived carbon out of carbon content for biodiesel and glycerol mixture,

0.09 : weight fraction of glycerol out of biodiesel and glycerol mixture,

0.91 : weight fraction of biodiesel out of biodiesel and glycerol mixture,

0.391: carbon content of glycerol,

0.861: carbon content of biodiesel (assumption: carbon content of biodiesel is the same as that of diesel) 3

WCO2,6k-III = 2.82 × 0.043 × 0.91 / 0.09 = 1.23 kg CO2/kg glycerol

where WCO2,6k-III : CO2 reduction amount per unit weight of produced glycerol as a biodiesel-waste,

0.09 : weight fraction of glycerol out of biodiesel and glycerol mixture,

0.91 : weight fraction of biodiesel out of biodiesel and glycerol mixture

Thus, the CO2 reduction amount per unit weight of produced KIE-6-k is

WCO2,6k-IV = 1.23 / 1000 × 2.18 = 0.0027 kg CO2/g KIE-6-k

where WCO2,6k-IV : CO2 reduction amount per unit weight of produced KIE-6-k,

2.18 : weight of glycerol required for preparing 1 g of KIE-6-k

**3.3. Calculation of net CO2 reduction during synthesis of KIE-6-k**

WCO2,6k-V = WCO2,6k-I - WCO2,6k-IV = 0.0031 – 0.0027 = 0.0004 kg CO2/g KIE-6-k

where WCO2,6k-V : net CO2 reduction during synthesis of KIE-6-k

CO2 reduction rate = 0.0027/0.0031 × 100 = 87.1 %

Accordingly, when glycerol is used as a pore-forming agent for mesoporous silica KIE-6-k (the weight ratio of glycerol to silica nanoparticles is 2.1), CO2 emission during preparation of KIE-6-k is expected to be reduced by about 87.1 %.

**4. Investigation on CO2 reduction effect through a green synthetic methodology of mesoporous carbon KIE-7 (Case I): silica etching with NaOH solution**

**4.1. Calculation of CO2 emission during synthesis of KIE-7-b**

When the total weight of G-SN nanocomposite sol is set to 27.28 g, the nanocomposite sol consists of 2.52 g of silica nanoparticles, 15 g of distilled water, 4.26 g of ethanol, and 5.5 g of glycerol. The first step of KIE-7-b synthesis is drying and precarbonization of the nanocomposite sol at 150 oC. The energy required for thermal treatment of G-SN nanocomposite sol (27.28 g) at 150 oC can be calculated as below.

Q150 = (Wsn × Csn × (150-25) + Wg × Cg × (150-25) + Ww × Cw × (100-25) + Ww × Qw

+ We × Ce × (78.37-25) ­+ We × Qe) + (Wsn × Csn + Wg × Cg) × HL × t150

= (2.52 × 0.22 × (150-25) + 5.5 × 0.58 × (150-25) + 15 × 1 × (100-25) + 15 × 539

+ 4.26 × 0.54 × (78.37-25) ­+ 4.26 × 229) + (2.52 × 0.22 + 5.5 × 0.58) × 100 × 12

= 15,269.6 cal

where Q150 : energy required for thermal treatment of G-SN nanocomposites at 150 oC (cal),

Wsn, Wg, Ww, We : weight of silica nanoparticles, glycerol, water, and ethanol (g),

Csn, Cg, Cw, Ce : specific heat of silica nanoparticles, glycerol, water, and ethanol (cal/g.oC),

Qw, Qe : heat of vaporization of water, and ethanol (cal/g),

HL : heat loss rate of an electric furnace (assumption: 100 oC/h),

t150 : thermal treatment time at 150 oC (12 h)

Meanwhile, we measured that the weight of G-SN nanocomposites was reduced from 27.28 g to 4.56 g after the thermal treatment at 150 oC, and 4.56 g of PG-SN nanocomposites comprises 2.52 g of silica nanoparticles and 2.04 g of precarbonized glycerol. For carbonization of glycerol, the PG-SN composites were carbonized for 3 h under nitrogen atmosphere at 600 oC. If we assume that precarbonized glycerol is converted to carbon without weight loss by carbonization at high temperature, we can conservatively calculate the energy required for carbonization of PG-SN nanocomposites at 600 oC as below.

Q600 = (Wsn × Csn × (600-25) + Wc × Cc × (600-25)) + (Wsn × Csn + Wc × Cc) × HL × t600

= (2.52 × 0.22 × (600-25) + 2.04 × 0.17 × (600-25)) + (2.52 × 0.22 + 2.04 × 0.17)

× 100 × 3

= 788.6 cal

where Q600 : energy required for carbonization of PG-SN nanocomposites at 600 oC (cal),

Wsn, Wc : weight of silica nanoparticles, and carbon (g),

Csn, Cc : specific heat of silica nanoparticles, and carbon (cal/g.oC),

HL : heat loss rate of an electric furnace (assumption: 100 oC/h),

t600 : carbonization time at 600 oC (3 h)

After carbonization at 600 oC, the weight of PG-SN nanocomposites decreased from 4.56 g to 2.71 g, and 2.71 g of the carbon-silica nanoparticle (C-SN) nanocomposite is composed of 2.52 g of silica nanoparticles and 0.19 g of carbon. If we assume that the C-SN nanocomposites are etched via 5 wt% NaOH solution treatment for 6 h at 75 oC in an oil bath and the NaOH solution comprises 75 g of water, 59.4 g of ethanol, and 6.6 g of NaOH, the energy required for etching of C-SN nanocomposites (2.71 g) can be calculated as

Qetch = (Wsn × Csn × (75-25) + Wc × Cc × (75-25) + Ww × Cw × (75-25) + We × Ce × (75-25) ­+ Wn × Cn × (75-25)) + (Wsn × Csn + Wc × Cc + Ww × Cw + We × Ce+ Wn × Cn) × HL × tetch

= (2.52 × 0.22 × (75-25) + 0.19 × 0.17 × (75-25) + 75 × 1 × (75-25) + 59.4 × 0.54 × (75-25)+ 6.6 × 0.78 × (75-25)) + (2.52 × 0.22 + 0.19 × 0.17 + 75 × 1 + 59.4 × 0.54 + 6.6 × 0.78) × 100 × 6

= 73,327 cal

where Qetch : energy required for etching of SN/C nanocomposites (cal),

Wn : weight of NaOH (g),

Cn : specific heat of NaOH (cal/g.oC),

HL : heat loss rate of an oil bath (assumption: 100 oC/h),

tetch : etching time (6 h)

If we ignore the thermal efficiency of the furnace and oil bath, and the energy required for heating air and silicone oil inside the furnace and oil bath, the total energy consumption and CO2 emission per 1 g of prepared KIE-7-b can be conservatively calculated as below:

Q = (Q150 + Q600 + Qetch)/WKIE-7-b = (15,269.6 + 788.6 + 73,327.0)/0.19

= 470,448.4 cal/g KIE-7-b

where WKIE-7-b : weight of synthesized KIE-7-b after etching

WCO2,7b-I = 470,448.4 / 1000 × 0.001163 × 0.424 = 0.232 kg CO2/g KIE-7-b

where WCO2,7b-I : total CO2 emission during synthesis of KIE-7-b [Case I],

0.001163: unit conversion factor (kWh/kcal),

0.424: CO2 emission coefficient (kg CO2/kWh)

**4.2. Calculation of CO2 reduction by biodiesel-waste glycerol used for synthesis of KIE-7-b**

In previous section, CO2 reduction amount per unit weight of produced glycerol as a biodiesel-waste has been already calculated to be 1.23 kg CO2/kg glycerol. Thus, the CO2 reduction amount per unit weight of produced KIE-7-b can be readily estimated as below.

WCO2,7b-II = 1.23 / 1000 × 28.95 = 0.036 kg CO2/g KIE-7-b

where WCO2, 7b-II: CO2 reduction amount per unit weight of produced KIE-7-b [Case I],

1.23 : CO2 reduction amount per unit weight of produced glycerol as a biodiesel-waste,

28.95 : weight of glycerol required for preparing 1 g of KIE-7-b

**4.3. Calculation of net CO2 emission during synthesis of KIE-7-b**

WCO2,7b-III = WCO2,7b-I - WCO2,7b-II = 0.232 – 0.036 = 0.196 kg CO2/g KIE-7-b

where WCO2,7b-III : net CO2 emission during synthesis of KIE-7-b [Case I]

CO2 reduction rate = 0.036/0.232 × 100 = 15.5 %

Accordingly, when glycerol is used as a carbon precursor of mesoporous carbon KIE-7-b (the weight ratio of glycerol to silica nanoparticles is 2.1) and silica etching is conducted with NaOH solution at relatively high temperature, CO2 emission during preparation of KIE-7-b is expected to be reduced by about 15.5 %.

**5. Investigation on CO2 reduction effect through a green synthetic methodology of mesoporous carbon KIE-7 (Case II): silica etching with HF solution**

**5.1. Calculation of CO2 emission during synthesis of KIE-7-b**

Q150, Q600, and WKIE-7-b are the same as those of Case I. However, etching procedures with HF solution are conducted at room temperature. Thus Qetch can be ignored. The total energy consumption and CO2 emission per 1 g of prepared KIE-7-b can be conservatively calculated as below:

Q = (Q150 + Q600 + Qetch)/WKIE-4 = (15,269.6 + 788.6 + 0) / 0.19 = 84,516.8 cal/g KIE-7-b

WCO2,7b-IV = 84,516.8 / 1000 × 0.001163 × 0.424 = 0.042 kg CO2/g KIE-7-b

where WCO2, 7b-IV: total CO2 emission during synthesis of KIE-7-b [Case II],

0.001163: unit conversion factor (kWh/kcal),

0.424: CO2 emission coefficient (kg CO2/kWh)

**5.2. Calculation of CO2 reduction by biodiesel-waste glycerol used for synthesis of KIE-7-b**

CO2 reduction amount per unit weight of produced KIE-7-b for Case II is the same as that for Case I (WCO2,7b-II).

WCO2,7b-V = WCO2, 7b-II = 0.036 kg CO2/g KIE-7-b

where WCO2, 7b-V : CO2 reduction amount per unit weight of produced KIE-7-b [Case II]

**5.3. Calculation of net CO2 emission during synthesis of KIE-7-b**

WCO2,7b-VI = WCO2,7b-IV - WCO2,7b-V = 0.042 – 0.036 = 0.006 kg CO2/g KIE-7-b

where WCO2,7b-VI : net CO2 emission during synthesis of KIE-7-b [Case II]

CO2 reduction rate = 0.036/0.042 × 100 = 85.7 %

Accordingly, when glycerol is used as a carbon precursor of mesoporous carbon KIE-7-b (the weight ratio of glycerol to silica nanoparticles is 2.1) and silica etching is conducted with HF solution at room temperature, CO2 emission during preparation of KIE-7-b is expected to be reduced by about 85.7 %.

**6. Supporting references**

1. E.A. Nanaki and C.J. Koroneos, *J Clean. Prod.* 2012, **20**, 14-19.

2. Wikipedia < http://en.wikipedia.org/wiki/Biodiesel >

3. Wikipedia < http://en.wikipedia.org/wiki/Diesel_fuel >
